# Role of Flavonoids in the Interactions among Obesity, Inflammation, and Autophagy

**DOI:** 10.3390/ph13110342

**Published:** 2020-10-26

**Authors:** María José García-Barrado, María Carmen Iglesias-Osma, Elena Pérez-García, Sixto Carrero, Enrique J. Blanco, Marta Carretero-Hernández, José Carretero

**Affiliations:** 1 Department of Physiology and Pharmacology, Faculty of Medicine, University of Salamanca, 37007 Salamanca, Spain; mcio@usal.es (M.C.I.-O.); elenaperez955@usal.es (E.P.-G.); 2 Laboratory of Neuroendocrinology, Institute of Neurosciences of Castilla y León (INCyL), and Laboratory of Neuroendocrinology and Obesity, Institute of Biomedical Research of Salamanca (IBSAL), University of Salamanca, 37007 Salamanca, Spain; ejbb@usal.es (E.J.B.); jcar@usal.es (J.C.); 3 Surgery Service of the University, Hospital of Salamanca, 37007 Salamanca, Spain; scarrero55@gmail.com; 4 Department of Human Anatomy and Histology, Faculty of Medicine, University of Salamanca, 37007 Salamanca, Spain; martataes@gmail.com

**Keywords:** autophagy, obesity, inflammation, flavonoids, quercetin

## Abstract

Nowadays, obesity is considered as one of the main concerns for public health worldwide, since it encompasses up to 39% of overweight and 13% obese (WHO) adults. It develops because of the imbalance in the energy intake/expenditure ratio, which leads to excess nutrients and results in dysfunction of adipose tissue. The hypertrophy of adipocytes and the nutrients excess trigger the induction of inflammatory signaling through various pathways, among others, an increase in the expression of pro-inflammatory adipocytokines, and stress of the endoplasmic reticulum (ER). A better understanding of obesity and preventing its complications are beneficial for obese patients on two facets: treating obesity, and treating and preventing the pathologies associated with it. Hitherto, therapeutic itineraries in most cases are based on lifestyle modifications, bariatric surgery, and pharmacotherapy despite none of them have achieved optimal results. Therefore, diet can play an important role in the prevention of adiposity, as well as the associated disorders. Recent results have shown that flavonoids intake have an essential role in protecting against oxidative damage phenomena, and presents biochemical and pharmacological functions beneficial to human health. This review summarizes the current knowledge of the anti-inflammatory actions and autophagic flux of natural flavonoids, and their molecular mechanisms for preventing and/or treating obesity.

## 1. Introduction

Numerous polyphenolic compounds have been identified in plants of which flavonoids are the most important group. More than 5000 natural flavonoids have been described and compounds belonging to this group continue to be identified today. They are broadly distributed in fruits, vegetables, seeds, nuts, stems, and flowers, as well as in products derived from them, such as, wine, juices, or beer, which are important constituents of the human diet. The name flavonoids came from the Latin word “flavus,” which means yellow. They contain a fenibenzopiran structure and are secondary metabolites of fungi and plants [1].

**Figure 1:**
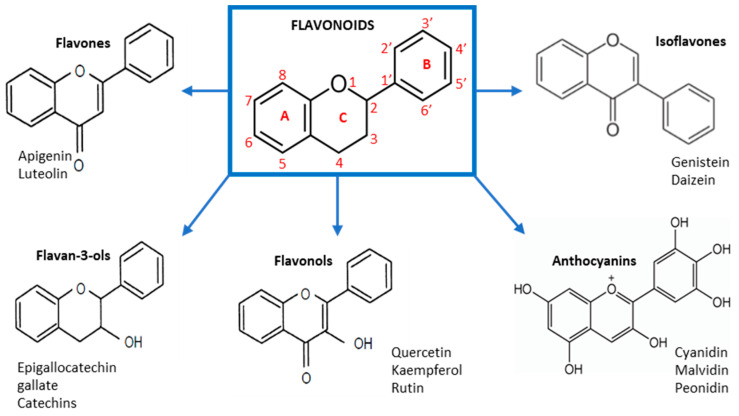
The chemical structures of some of the most relevant flavonoids belonging to the above-mentioned classes.

The flavonoid chemical structure at its simplest level has a characteristic C6-C3-C6 structure. Specifically, it contains two aromatic rings (also called rings A and B) that are joined by a chain of three carbons, producing an oxygenated heterocycle (ring C) [1]. At the base of its heterocycle structure and depending on the degree of saturation and the substitutes of the C-ring, the flavonoids are classified into several classes, including flavones, flavonols, flavanons, flavan-3-oles, isoflavones, and anthocyanins. Within these groups, flavonoids are distinguished by the substitution of A and B ring, and the type of substitution in the different hydroxyls of the molecule (Figure 1) [2]. This structure supplies them excellent chelation properties of iron and other transitional metals, which provide a great antioxidant capacity [1]. Therefore, they play an essential role in protecting against oxidative damage phenomena, and presents biochemical and pharmacological functions beneficial to human health. 

Bioactive compounds have been and continue to be widely studied, and have shown not only an essential role as nutrients, but also have an extensive variety of biological actions. These actions include antioxidant and anti-inflammatory activity, hepatoprotective effect, antibacterial activity, antiviral activity, anticancer activity, and antidiabetic activity, among others [3,4]. On the other hand, flavonoids are present as fundamental ingredients in healthy food preparations, such as dietary supplements, and are even included in cosmetic preparations. The safety of bioactive compounds and their bioavailability in the human organism are also being broadly explored to know the benefits of new natural sources on health [5,6]. Recent results have shown the beneficial effects of these compounds on cardiovascular disorders, neurodegenerative diseases, some cancers, and osteoporosis, and the inverse relationship between flavonoid intake and the risk of obesity and diabetes has been proposed [2,7].

## 2. Methodology

This study is a literature descriptive review. The data sources we have used were databases as follows: PubMed, Springer, ScienceDirect, Wiley, and Scopus. The main inclusion criteria were the terms “flavonoids,” “obesity,” “anti-inflammatory effect,” “autophagy.”. Secondary searches were performed adding the term “in vitro,” “in vivo,” “review,” or “clinical trial” to the former terms. Due to the wide literature related, only the most relevant papers were selected considering the quality of the study, quality of the journal, most recent years of publication, and the diversity of mechanisms and models assayed. Given the vast number of flavonoid compounds, this review focused, especially on those compounds most studied in obesity, like quercetin and epigallocatechin gallate (ECGC). The exclusion criteria were lack of relation with the topic according to title and abstract evaluations, incomplete data, and a language other than English or Spanish. Finally, 199 studies met the selection criteria and were included in our study.

## 3. Biosynthesis and Bioavailability

Most flavonoids exist in their natural media in the form of glycosidic derivatives, and sugar (glucose) is the most common among them, although they may also be methylated or present as free aglycones. Their effects on human health partly depend on multiple factors, including their chemical structure and solubility, the interaction with endogenous components, membrane transporters, and/or enzymes that modulate certain pathways and even with the gut microbiota. All contribute to the variability in the bioavailability of the different flavonoids [8,9]. Nutritional flavonoids are absorbed into the gastrointestinal tract where they are hydrolyzed enzymatically by bacterial enzymes to release the aglycones and, thus, improve their absorption in the small intestine. The aglycones generally have better bioavailability and earlier absorption than glycosides because of better membrane interactions [10].

Those unabsorbed flavonoids are metabolized by colon bacteria to give smaller compounds that are transported to the circulatory system [11,12]. However, they can also be prevented from reaching the circulatory system by interacting with glycoprotein P, widely expressed throughout the gastrointestinal tract [13]. Once absorbed, they are primarily biotransformed in the liver and intestine by hydrolysis, binding, cracking, and oxidation biotransformation reactions, or in the colon by phase II biotransformation reactions, in which microorganisms degrade non absorbed flavonoids forming gluconic acid conjugates, sulfates, and methides which may also occur in the liver [14]. Water-soluble conjugates can be excreted by urine or bile [8,15]. Pharmacokinetic studies have shown that the half-life of a typical flavonoid ranges from 1–2 h [16].

Quercetin, catechins, and kaempferol are the most abundant flavonoids in the diet belonging to species of vegetables such as garlic, onions, lettuce, and tomato, among others. The content and composition in each of them can be found in numerous government databases or those developed by various agencies. It is worth highlighting the database developed in the United States by the USDA [17] and in Europe EuroFIR developed eBASIS [18], and ePlantLIBRA [19]. Besides, information about these compounds can be found in The Dietary Supplement Label Database (DSLD) [20], and also in the following links of FAO/INFOODS [21], Phenol-Exporer [22], and AESAN [23] that include food and nutrients composition.

Quercetin, a widely studied flavonol, is principally absorbed into the gut cells. The beta-glucosidases enzymes are responsible for hydrolyzing the glucosides in the small intestine to the aglycone form, much of which is then absorbed. Quercetin can be metabolized to glucuronide, methylated and sulfated derivatives. Its metabolites are eliminated mainly in the gut, and a small portion is excreted in the urine by the kidneys [24]. The metabolites have a longer half-life than those of other compounds such as catechin showing differences in their bioavailability [25]. Recent studies have pointed to the important role of the gut microbiota in the absorption of quercetin, as it promotes its transformation into smaller and more easily absorbable compounds [26]. 

The average dietary intake of flavonols has been calculated at 20–35 mg/day; however, the amount ingested with the diet depends on many variables such as variations in plant composition depending on its genetics, dietary habits, and how the food is prepared. At present, the average consumption of quercetin is considered to range from 6–18 mg/day in the United States, China, and Europe [27], even though other authors raise their consumption to 50 mg/day [28]. In addition, it should be kept in mind that purified quercetin is also commercialized in the EU and USA, and is available in capsule form or oral solutions.

## 4. Obesity and Health

Obesity has become one of the global issues for public health, and currently is reaching up to 39% of overweight and 13% obese (WHO) adults. An estimated 41 million children under five-years-old and more than 340 million children and adolescents among 5 to 19 are overweight or obese [29]. The WHO has identified obesity as one of the main health problems of the 21st century. It develops because of the imbalance in the energy intake/expenditure ratio which leads to excess nutrients and results in dysfunction of adipose tissue. This increased fat mass produces a raise in the number of adipocytes (hyperplasia) and/or their size (hypertrophy). Increased adiposity, mainly in visceral adipose tissue, is directly associated with cardiovascular pathologies, non-alcoholic fatty liver disease (NAFLD), insulin resistance, and type 2 diabetes, among others.

Adipose tissue, along with the classic triglyceride storage functions, plays an important role in physiological processes such as the development and growth of adipocyte and energy homeostasis. These functions on energy metabolism are regulated by adipocytokines secreted from adipocytes and including hormones (leptin, adiponectin, resistin, visfatin), cytokines (IL-6, TNFα), and other bioactive factors with specific biological functions. Furthermore, angiogenesis, extracellular matrix remodeling, immune response, steroid metabolism, as well as glucose homeostasis and insulin sensitivity, are processes where adipocytes participate actively [30,31]. The alteration of the different biological actions of adipose tissue involves the development of relevant syndromes, which entail great relevance. Therefore, a better understanding of obesity and preventing its complications are beneficial for obese patients on two facets: treating obesity, and treating and preventing the pathologies associated with it.

### 4.1. Obesity and Inflammation

Living beings have developed several defensive strategies against attacks on their health, among them, inflammation is one of the most effective and important. In situations of obesity, the main trigger for inflammation is excess nutrient consumption. Inflammation commonly seen in obese and overweight patients is described as meta-inflammation: chronic and low-grade inflammation response [30,32].

Adipocytes are the main cells of the fat tissue, but there are also other cellular elements from the vascular stroma, such as preadipocytes, endothelial cells, several immune cells, and fibroblasts. However, a disturbing infiltration of macrophages is observed in the adipose tissue of obese patients [33,34]. The hypertrophy of adipocytes and the nutrients excess trigger the induction of inflammatory signaling through various pathways, among others, an increase in the expression of pro-inflammatory adipocytokines, and stress of the endoplasmic reticulum (ER).

In this context, it is very important to keep in mind the inflammatory complex—inflammasome—represented by a set of proteins and Toll-like receptors (TLR), NOD-like receptors (NLRs, nucleotide-binding oligomerization domain-like receptors) among others, which activate inflammatory caspases and pro-inflammatory cytokines (e.g., NF-κB, IL-1β, IL-6). High-fat diets cause the activation of cytokine receptors and Toll-like receptors (TLRs). These, through chaperone molecules, activate the MAPK (mitogen-activated protein kinase) pathway and stimulate the translocation of NF-κB (nuclear factor-kappa-B) [30] and, in turn, promote the transcription of genes involved in the inflammatory response [19]. In obesity, the inflammasome NLPR3 (which belongs to NOD-like receptors) is widely identified [34].

The first relevant data in this situation are the high level of inflammatory cytokine TNFα (tumor necrosis factor-alpha) compared to lean individuals that produce low-grade inflammation [35]. This way, the low-grade inflammation induces the expression of stress markers such as IKK (inhibitor of nuclear factor kappa-β kinase), JNK (c-Jun N-terminal kinase), and PKR (protein kinase R) that alter the balance of immune cells favoring a pro-inflammatory atmosphere. The three kinases activate the transcription factors AP-1 (activator protein 1), NF-κB, and IRF (interferon regulatory factor) that regulate the genetic expression of inflammatory mediators [36]. These kinases, in turn, can inhibit insulin signaling by IRS-1 protein serine phosphorylation and negatively regulate the eIF2 (initiation factor of eukaryotic translation 2α). Moreover, they not only inhibit insulin action through their action on IRS-1 proteins but also increase the expression of pro-inflammatory cytokines, and exacerbate inhibitory signaling of metabolic pathways.

On the other hand, MCP-1 (monocyte chemoattractant protein-1) is released and through its chemotactic properties in cells, favors the infiltration of macrophages into adipose tissue. It has been shown that the levels of MCP-1 in obese mice versus lean mice are higher, and its levels are increased when adipocytes are co-cultivated with macrophages [37,38]. Infiltration of macrophages into adipose tissue and subsequent release of TNFα [39], make insulin signaling difficult and stimulate lipolysis of triglycerides into free fatty acids in adipocytes. Adipocytes treated with TFNα have been seen to decrease glucose uptake [40]. In the chronic low-grade inflammation, that is closely related with insulin resistance, other pro-inflammatory cytokines including interleukin-6 (IL-6), interleukin-1 (IL-1), and C-reactive protein [41] are also involved. This way it perpetuates inflammation of adipose tissue and insulin resistance that is usually associated with adiposity [42]. Contradictorily to the classic model of inflammation in other tissues, the meta-inflammation that occurs in obesity is associated with a decrease in the metabolic rate.

Similarly, inflammation of adipose tissue can negatively control the expression of the PPARγ receptor (peroxisome proliferator activated receptor γ), and impair the normal adipogenesis process [43]. Additionally, fat expansion requires adequate vascularity to allow oxygen and nutrients to be supplied to tissues.

In obesity, excessive fat accumulation promotes the saturation of oxidative and storage pathways in adipocytes, producing in such cases a decrease in their reserve capacity and facilitating the flow of lipids to other tissues or organs resulting in the deposition of ectopic fat in visceral deposits, liver, and other cell types. This toxic response is called lipotoxicity [44] and involves changes in cell and extracellular matrix composition, increased number of immune cells infiltrating into adipose tissue, increased autophagy and apoptosis, as well as changes in adipose tissue protein mRNA expression patterns. Furthermore, many of the pathways by which excess fatty acids could be directed participate in insulin resistance and/or apoptosis and autophagy of fat cells [45].

### 4.2. Obesity and Autophagy

Autophagy is a biological degradation process perfectly designed and fundamental for the homeostasis of eukaryotic cells [46,47]. There are several types of autophagy, namely non-selective macroautophagy (here referred to as autophagy), selective autophagy, chaperone-mediated autophagy, and microautophagy [47,48]. Autophagy is defined as an event where cytoplasmic material—both long-life macromolecules and organelles—are captured in a double membrane vesicle called autophagosome that in a second step will fuse with the lysosome and finally those constituent components are degraded and/or recycled. A very large number of genes called Atg responsible for autophagic flow can participate in the complex cycle of autophagosome formation [49,50].

Although self-digestion does not seem beneficial to cells at first glance, it is considered a protective process as it provides signals for the elimination of apoptosis, genomic stability, and nutrient deprivation situations that provide resources to survive [51].

Basal autophagy is necessary for providing nutrients to maintain crucial cellular functions when the cells present deprivation of nutrients, while on the other hand, it favors the elimination of excess or damaged organelles, unfolded proteins and lipids in the overflow of nutrient. Therefore, it is not easy to discern the extent to which autophagy promotes or decreases cellular survival based on the cellular context and the environment [52].

Alterations in autophagy, either increases or decreases in it, have been shown to be involved in the pathogenesis of various illness, including cancer, neurological, cardiovascular, metabolic diseases such as obesity, diabetes mellitus, and aging [46,53,54]. Specifically, an altered (increased or suppressed) autophagy has been reported in genetically modified or diet-induced animal models of obesity [55] as well as in obese patients.

In situations of obesity or overweight, understanding the changes that occur in autophagy is a very complex process since it may depend on the nature and animal models used to study it, as well as the types of cells and tissues, the techniques used in the study, and many other variables (see Table 1). It is therefore difficult to interpret the results found when studying autophagy since in some cases they may seem incoherent. In general, all research advocates the fundamental role of autophagy in the development of obesity and the complications it generates. However, it is important to consider that the relationship between autophagy and obesity is not limited exclusively to its activity on adipose tissue [55]. It is established that food intake is regulated from the hypothalamus and is linked to autophagic signaling pathways including mTOR, AMPK, FOXO, and PI3K [56]. Therefore, dysregulation in hypothalamic autophagy can lead to excess food consumption, which is one of the situations that causes obesity. In periods of starvation, circulating free fatty acids increase and by themselves can activate the AMPK/ULK1 protein complex and the cell signaling cascade to initiate autophagy, and this increases the expression of the aguti-related peptide (AgPR) that stimulates food intake and the development of obesity [57].

Autophagy has been described that regulates adipose mass in different mouse models by blocking adipogenesis and expression of its markers [82]. In other cases, it is related to hepatic steatosis [83], as well as the production and release of adipokines in adipocytes [84].

On the other hand, it should be noted that research in the adipose tissue of obese people showed higher expression of autophagy genes ATG5-12, and the proteins that form a stable association with the membrane of autophagosomes LC3I(A), and LC3II(B). Studies on the different types of adipose tissue, whether visceral or subcutaneous, revealed interesting results. Following the analysis by Western blot of LC3-II expression, ATG5-12 protein complex, mTOR, and transcriptional regulation of ATG, it was confirmed that autophagic flow was increased in both visceral and subcutaneous adipose tissue of obese patients (with and without DM2) compared to control individuals. These results suggested an improvement in autophagic clearance and/or increased accumulation of autophagosomes. Another autophagy marker, Beclin protein, was increased in obese patients versus the controls prior to surgery. ATG12 mRNA expression was positively matched up with the degree of obesity, the presence of visceral fat, and adipocyte hypertrophy [62], confirming increased autophagic flow. However, accumulated autophagosomes may indicate an obstruction in the autophagy or an increase in autophagosome formation [48]. Other studies have seen that the elimination of some important autophagy-related genes (ATG13, ULK2, RB1CC1, ATG5, ATG7, BECN1) may inhibit adipocyte differentiation [85]. The role of mTOR in stress regulation and autophagy has also been documented by suggesting that the mechanism of activation of autophagy in obese patients could be related to decreased mTOR expression [64].

Conversely, some authors propose that obesity can inhibit autophagy. Accordingly, mitochondrial and endoplasmic reticulum oxidative stress, and the accumulation of toxic substances stored but not eliminated, could be responsible for the insulin resistance triggering, among others [65]. Soussi et al. described the reduction of autophagic flow in adipocytes of subcutaneous tissue in obese subjects [86]. The latter is also in concordance with the results reported in the state of restriction of nutrient intake, where an increase in autophagy activity is observed in obese people, and this increase in the level of autophagy was related to an improvement in insulin sensitivity [87]. Such outcomes emphasize that in obesity, the loss of autophagy control leads to the alterations in glucose homeostasis, and the consequences that this entails.

It has been found that the increase in transcriptional factor E2F1 in obese adipose tissue is related to the expression of the *ATG* genes, mainly with those that participate in the later stages of the autophagic process such as ATG12, LC3-II, and DRAM1 [88,89]. E2F1-deficient adipocyte cells exposed to inflammatory cytokines showed less activation of autophagy [61,90]. Interestingly, E2F1 induction in adipose tissue appears simultaneously to inflammatory activation. This fact suggests that autophagy activation by E2F1, may act as a protective mechanism against inflammation linked to obesity [61,90]. This is coherent with the idea that there is a simultaneous correlation between the regulation of the autophagy and the inflammation. Many of cytokines or adipokines liberated during low-grade inflammation cause autophagy, which is a relevant mechanism for removing invasive pathogens.

Based on the current knowledge, the role of autophagy in obesity remains complex. LC3II marker data can be misleading regarding autophagic flow, since it depends on the model of study of obesity, the cell composition of the tissue studied (adipocytes, fibroblasts, macrophages, or other immune cells), the existence of comorbidities, and even the experimental techniques used to evaluate it [55].

As stated in a previous review, there are still many lacks in explaining the role of autophagy in obesity [55]. In this regard, it is in doubt whether the autophagy of adipose tissue is improved or suppressed in obesity, or whether the alteration of autophagy is a direct consequence of increased lipid content or a compensatory response to contribute to recycling the excess lipids. It is unknown whether it acts as a mechanism for removing lipid drops (lipophagia), or for the biogenesis of lipid drops, or both (depending on the type of cells). Furthermore, increased autophagy in adipose tissue or other tissue in obese patients does not necessarily imply that autophagy triggers obesity and the specific complications that would be developed.

### 4.3. Inflammation and Autophagy in Obesity

As described in the section of obesity and inflammation, adipokines released during low-grade inflammation (leptin, resistin, adiponectin, visfatin), as well as other classic cytokines (TNFα, IL-6, IL-1, MCP-1), promote different autophagy responses, like stimulation (e.g., adiponectin, leptin, IFNγ) or inhibition (e.g., interleukin) depending on the type of tissue [41,62,91].

In this sense, IL-6 has shown contradictory results in non-fatty tissues, specifically bronchial epithelial cells, where the IL-6 overexpression decreases the autophagy [92], while in mouse myeloid cells and pancreatic tumor cells this interleukin favors autophagic flow [93]. In hippocampal neurons and renal cells, IL-1β and TGF-β induce autophagy, possibly by inhibiting mTOR and even modulating the JNK, Smad, and TAK1 (mitogen-activated protein kinase kinase kinase 7) signaling pathways [94,95]. In state of nutrient deprivation, other anti-inflammatory cytokines can suppress autophagy, such as IL-4, IL-10, and IL-13. These cytokines trigger this process by stimulating the PI3K/Akt signaling pathway. Another cytokine involved in the elimination of pathogens, IFN-γ, has shown an increase in autophagic flux dependent on the activation of signal transducers STAT6 (signal transducer and activator of transcription 6), and independent of the Akt pathway in macrophages [96]. Pro-inflammatory cytokines have been shown to impair the storage of triglycerides through the induction of autophagy in the adipose tissue of obese patients [97].

At the same time, other stress-signaling pathways such as those of the transcription factor NF-κB or the AP-1 complex are also activated and generate different regulatory responses of autophagy. The inflammatory signaling, especially that activated by NF-κB and the inflammasome, depends on oxidative stress and ROS. These same factors also regulate autophagic gene transcription and, thus, autophagic flux. A general analysis of gene expression profiles has been carried out in the omental adipose tissue of obese patients. The results have shown that 34 lysosomal/autophagic genes are upregulated. These results confirm an increase in autophagy activity in obese adipocytes. The interpretation of this fact has been attributed to the increase in pro-inflammatory cytokines released from macrophages [97]. The same authors confirmed the upregulation of lysosomal/autophagic genes in murine 3T3-L1 adipocytes treated for 24 h with TNFα, and even the autophagy marker SQSTM1/p62 was also elevated.

Adipocytes are surrounded by structural proteins belonging to the PLIN family, which are key to their functioning. Of the three identified (PLIN1, PLIN2, and PLIN3), perilipin1 (lipid droplet-associated protein, PLIN1) is the one that is widely expressed in adipocytes, while the other two are ubiquitous. Some authors have confirmed that these proteins (mainly PLIN2 and 3) can be substrates of chaperone-mediated autophagy (CMA), and their degradation through CMA is prior to lipolysis [98]. Other more recent research has shown that TNFα acts by promoting the degradation of perilipin 1 (PLIN1). This process is carried out directly through ubiquitination induced by the mediator of autophagy SQSTM1/p62. Thus, TNFα may play a decisive role in the selective degradation of PLIN1 through the induction of autophagy [97]. With the data known so far, it is not ruled out that in the adipose tissue of the obese in which a low degree of inflammation has been shown, autophagy may play an important role in lipid metabolism. Inflammatory stress induces autophagy, and this increases lipolysis and ectopic lipid deposition. These facts favor the appearance of other pathologies associated with obesity such as insulin resistance.

On the other hand, NLRP3 inflammasome is a determining marker of obesity-associated inflammation [55], and is also associated with autophagy, as it is well reported that autophagy (principally mitophagy) controls NLRP3 levels [99]. Li et al. have found that mTOR can activate the inflammasome NLRP3 via mitochondrial reactive oxygen species. In turn, the inhibition of mTOR can inhibit inflammasome NLRP3 [100]. As a negative autophagy regulator, mTOR inhibition can also trigger autophagy [64]. Therefore, mTOR is a connection point that joins inflammation with autophagy. Likewise, inflammasome could serve as another link between inflammation and autophagy in obesity.

It is considered that autophagy protects against inflammation through the elimination of pathogens or intracellular organelles, and the suppression of pro-inflammatory complexes. Studies linked to the presence of pathogens and autophagy, tried to establish the connection with the “pattern recognition receptors” TLR. The results are discrepant, since in macrophages, the stimulation of autophagy induced by the activation of TLR4 and TLR7 is related to the adapter MyD88 and TRIF [101,102,103]. TRIF and/or Myd88 adapter proteins would reduce the binding of beclin1 to the inhibitory protein Bcl2, leading to the activation of autophagy [104].

**Figure 2:**
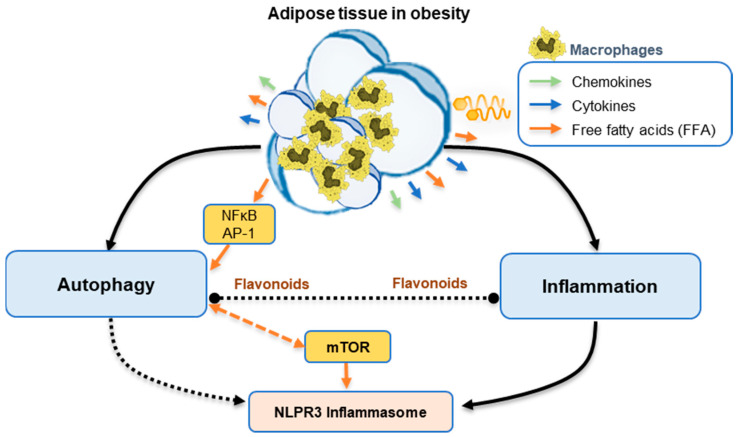
Scheme of the relationship between inflammation and autophagy in obesity.

Other transcription factors can also modulate inflammation and autophagy, this is the case of those belonging to the nuclear receptor family, such as the peroxisome proliferator-activated receptor α (PPARα), PPARγ, and the farnesoid receptor activated Xa (FXR) [105,106], as well as the transcription factor EB (TFEB). The first two, PPARα and FXR, have opposite effects on autophagic gene transcription, although both have been reported to compete for the same binding site in autophagy genetic promoters [107]. Likewise, in an inflammatory state, TFEB is an important regulator of autophagy and lysosomal genetic expression [93]. However, while there is a broad consensus to establish a reciprocal relationship between inflammation and autophagy regulation, scientific bases have not been established that reveal the intimate mechanisms to clarify what is the role that autophagy plays in the meta-inflammation that occurs in obesity (Figure 2) [93].

## 5. What is the Role of Flavonoids in the Autophagy Process and the Prevention of Obesity?

Considering the data presented, it is essential for further knowledge and understanding to provide new therapeutic strategies for the treatment of metabolic diseases such as obesity and others. Hitherto, therapeutic itineraries in most cases are based on lifestyle modifications, bariatric surgery, and pharmacotherapy, despite none of them have achieved optimal results. The diet can play an important role in the prevention of adiposity, as well as the associated disorders. However, the role of nutrients in autophagy regulation in obesity and their comorbidities is not clear yet.

### 5.1. Flavonoids and Autophagy

Flavonoids daily intake associated with the consumption of fruits and vegetables, and their ability to modulate cell signaling has been shown to be useful for health [108]. Recent results have shown beneficial actions in cardiovascular and neurodegenerative diseases, some types of cancer, osteoporosis, and the inverse relationship between anthocyanin and flavonoid intake, and the risk of obesity and diabetes has been proposed [7,109,110].

In recent years, one of the most investigated lines with flavonoid compounds has focused mainly on their anti-tumoral and anti-inflammatory properties, and their link with inflammation and autophagy, as shown by numerous data [108,111,112,113,114]. However, the study of these products is extraordinarily complicated because of the enormous number of identified compounds.

The anthocyanins are attributed to have diverse and controversial biological actions like antioxidant, anti-inflammatory, anti-diabetic properties, as well as inducing the arrest of the cell cycle and stimulating apoptosis or autophagy of cancer cells [111]. Likewise, quercetin (a flavonol) has also shown effects similar to those described for anthocyanins in tumor cells [111,115].

The beneficial effects of some flavonoids in oncological pathology are generally associated with their activity on autophagy and non-apoptotic cell death. Many reports have shown that flavonoids can stimulate autophagy in vivo and in vitro as a result of their antioxidant activity in the mitochondrial-endoplasmic reticulum and proteasome [116].

In cancer cell lines, as in the case of hematological cancer cells, the antitumor effect of flavonoids depends on their origin, whether it is myeloid, lymphoid, or erythroid. Apigenin, chrysin, and luteolin have been shown to have favorable properties in the treatment of cervical cancer, whereas luteolin and kaempferol can be included as good candidates for the treatment of gastric and ovarian cancer, respectively [117]. In contrast, in the breast and prostate cancer cells the toxic effects of flavonoids are greatly associated with the expression of hormonal receptors. In human pancreatic cancer cells, wogonin induces autophagy by activating beclin-1/PI3K and reactive oxygen species [118]. Other studies realized in chronic myeloid leukemia cells and K562 cells, show that curcumin can induce autophagy and apoptosis [119], and quercetin induces generalized autophagy in colon epithelial cancer cells, leading to cell cycle arrest and initiation of apoptosis [120].

Some flavonoids, like quercetin, epigallocatechin gallate (EGCG), and apigenin, showed antiproliferative effects by upregulating the expression of beclin-1, LC3-II, and forms of Atg in cells of hepatocellular carcinoma (HCC) [115]. In the same model, another flavonoid, apigenin was tested in the presence of the autophagy inhibitor 3-methyladenine (3-MA) and avoided the tumor growth [121]. Galangin suppressed proliferation, and induced apoptosis and autophagy (Li et al., 2016), a fact also demonstrated in glioblastomas and HepG2 cells where it induced autophagy, improved the binding of SIRT1-LC3, and reduced acetylation of endogenous LC3 [122]. In liver cancer cell, mulberry fruits extracts induced autophagy and apoptosis, and prevented hepatocarcinogenesis in vivo [123]. Specifically in HCC, the autophagy pathway seems to depend on two pathways, beclin-1-dependent and beclin-1-independent, and on the activation of other mediators such as the epidermal growth factor receptor (EGFR)/tyrosine-protein kinase Met (c-Met), NF-κB, MAPK, mTOR, and JNK kinases.

Flavonoids, on the other hand, have presented interesting properties by activating cardiac autophagy, hence they can be candidates for cardioprotective therapy. Thus, nobiletin, in an acute model of myocardial infarction in rats, restores the autophagy flow and exhibits a protective effect on the myocardium [124]. Apigenin can alleviate LPS-induced myocardial injury by modulating autophagic components such as lysosomal-associated membrane protein 1 (LAMP1), ATG5, p62, and TFEB [125]. The study realized with hypertensive patients treated with quercetin has showed a reduction in blood pressure [126]. The results were corroborated in diet-induced obesity rat models where the antioxidant activity of quercetin may also suppress the elevation of blood pressure [127]. Other natural compounds, like vitexin, rutin, EGCG, and luteolin, also showed positive effects on the regulation of autophagy in cardiovascular diseases [128,129,130,131]. Additionally, flavonoids such as naringenin, baicalin, EGCG, and quercetin have protective effects against bacteria or virus infection by promoting the activation of autophagy [132,133,134]. In endothelial cells treated with high glucose concentration, quercetin stimulates autophagy by negatively regulating p62 and the activation of beclin-1 and LC3-II. This reduces oxidative damage and improves the antioxidant defense system [135].

It is suggested that flavonoids could be encouraging candidates for the treatment of neurodegenerative diseases, linked with defective autophagy supported by their ability to modulate autophagy. In Alzheimer’s disease, quercetin can reduce abnormal protein aggregates such as β-amyloid peptides and hyperphosphorylated tau protein through autophagy pathway [136,137]. Furthermore, in rat primary neuron culture, EGCG also reduces phosphorylated tau protein by increasing mRNA expression of autophagy adapter proteins [138]. By in vivo Parkinson’s disease model, quercetin and baicalin augment the autophagic functions, and improve the neurotoxicity induced by rotenone [137,139].

Given the protective role of autophagy in the pathophysiology, it should be highlighted that autophagy modulation is an important target to regulate metabolic diseases [140], and the role of flavonoids deserves to be studied.

### 5.2. Flavonoids and Inflammation

There are multiple compounds of natural origin that have proven useful as anti-inflammatory agents. The anti-inflammatory effects of anthocyanins like cyanidin-3-glucoside, delphinidin-3-glucoside, and petunidin-3-glucoside have been demonstrated. These compounds inhibit NF-κB activity via mitogen-activated protein kinase (MAPK) pathways [141]. In bv2 microglial cells, anthocyanins downregulate the inflammatory responses induced by lipopolysaccharide by suppressing the NF-κB and AKT/MAPKs signaling pathways, and cyanidins inhibited cyclooxygenase enzyme activities. Antioxidant and anti-inflammatory activities of anthocyanins had effects on reducing lung inflammation in rats [142].

The work that studies the flavonoids inhibitory effect on the inflammation have focused mainly on the inhibition of inflammasome components expression such as NLRP3 and IL-1β, IL-18, and caspase-1. In cerebral ischemia models in rodents, anthocyanin from Myrica rubra and luteolin have been shown to have an inhibitory effect on the inflammasome through the TLR4/NF-κB/NLRP3 pathway [143,144]. Hyperin and zingerone have also shown to inhibit the NLRP3 inflammasome pathway in other models of LPS-induced acute kidney injury [145,146]. In streptozotocin-treated rats, quercetin ameliorated the kidney injury via the suppressed renal NLRP3 inflammasome activation [147].

In studies carried out with quercetin, it was observed that it has a good antioxidant and regulatory capacity for signal transduction pathways such as NF-κB, MAPK, and AMPK, and even inhibits lipid peroxidation [148]. It is undoubting that quercetin may have anti-inflammatory effects by inhibiting cytokine release and maintaining mast cell stability [149,150]. In Table 2 summarizes the effects of flavonois in the adipose tissue. 

The low-grade inflammation that occurs in obesity can be modified by flavonoids. In the Table 2 summarizes the effects of flavonois in the adipose tissue. Several studies showed that quercetin can inhibit the inflammatory response of macrophages by activating the AMPK phosphorylation and the expression of sirtuin 1 (SIRT1) [151]. Besides, it reduces obesity-induced hepatic inflammation by promoting macrophage phenotype switching [152]. In vivo, quercetin supplementation for 18 weeks to mice decreases the number of macrophages in adipose tissue [153]. Furthermore, in the high-fat diet (HFD) animal model, EGCG suppresses Toll-like receptor 4 (TLR4) expression, (firmly associated with induced inflammation in obesity), decreases macrophage infiltration, and also insulin resistance [154]. Naringenin also inhibits macrophage infiltration of the JNK pathway [155].

Other studies have shown in the HFD mouse model that apigenin attenuated the levels of metabolic inflammation in the colon through the activation of the peroxisome proliferator activated receptor (PPARγ), the reduction of malondialdehyde, IL-1β, and IL-6 [156]. In the same model, specific derivatives of chalcone prevented HFD-induced cardiac and kidney injury through the MAPK and NF-κB signaling pathway [91,157]. Furthermore, other isoflavones as daidzein [158], luteolin [159], chrysin [160], and rutin [130,161] also showed anti-inflammatory activity. There are several studies suggesting that flavonoids can reduce the obesity-related chronic inflammation considering their anti-inflammatory effect, and so, alleviating obesity [162,163]. A recent cohort study has also shown that flavonoid intake is inversely linked with body mass index and the level of C-reactive protein that is produced in response to inflammation [164].

The anti-obesity potential associated with flavonoids is quite relevant and their regulatory effects have been observed on reducing food intake, reducing nutrient absorption, modulating adipogenesis and the life cycle of adipocytes, induction of thermogenesis and energy consumption, and the regulation of the intestinal microbiota, among others.

In the first in vitro studies carried out to determine the role of flavonoids on adipose cells [189], the role of up to 31 flavonoids was studied in lipolysis in isolated rat adipocytes. Interestingly, quercetin induced lipolysis in a dose- and time-dependent manner by increasing cyclic adenosine monophosphate (cAMP) levels and hormone-sensitive lipase (HSL) activity.

Subsequently, other studies in rat fat pads showed that the gene expression levels of fatty acid synthase (FAS) and the activity of acetyl-CoA carboxylase (ACC) were inhibited. To confirm these results, the suppression of lipogenesis has been observed in adipocytes by reducing the incorporation rate of fatty acids [190].

Another important fact to keep in mind is if flavonoids exert actions on the absorption of carbohydrates. Initially, the inhibitory effect of flavonoids on α-amylase was observed with equal potency to acarbose as the positive control [191]. If the activity of α-glucosidase is reduced, the absorption of excess glucose can be prevented to further control the levels of blood glucose. This is another way to prevent obesity and diabetes [192,193].

An important point of regulation by flavonoids is the adipogenesis. The molecular mechanism of adipogenesis involves cell cycle proteins, transcription factors, lipogenesis genes, and related enzymes activities. The preadipocytes, to become adipocytes, need to be stimulated by PPARγ and C/EBPβ. Although the expression of PPARγ depends on C/EBPβ and C/EBPδ. Treatment with quercetin reduces C/EBPα, PPARγ, and SREBP-1 expression, and hence suppresses the differentiation of preadipocytes to adipocytes. Furthermore, quercetin derivates also significantly down-regulated expression of adipogenic genes highly expressed at the end stage of adipogenesis, as lipoprotein lipase (LPL) and adipocyte FA-binding protein (aP2) [165], and similar effects have been identified with catechin 3-gallate (CG) y EGCG. In 3T3-L1 preadipocytes, quercetin induces anti-adipogenesis activity by activating the AMPK signaling pathway while the quercetin causing apoptosis of mature adipocytes was mediated by modulation of the ERK and JNK pathways, which play pivotal roles during apoptosis [166].

Quercetin derivatives supplementation in rats improved both dyslipidemia and glucose tolerance. In the same model, the activation of the genes related with the beta-oxidation reduces the accumulation of hepatic lipid. Besides, in a model of a high-fat diet, quercetin lessened weight gain as well as the increase in adipocyte size [168]. Adipokines related to obesity and adipose tissue dysfunction can significantly decrease the levels of other adipokines (e.g., ANGPTL4, adipsin, and PAI-1), as well as glycolysis-associated enzymes (ENO2, PFKP, and PFKFB4) in SGBS adipocytes treated by quercetin [167].

Thermogenesis is a decisive process that is involved in the expenditure of energy in the body. It prevents fat accumulation, thereby promoting satiety, decreasing hunger, and increasing fat oxidation [194]. The adipose tissue is responsible for this process since it regulates the energy balance through the brown adipose tissue (BAT). In brown and beige adipocytes, AMPK/PGC1α signaling is an essential regulator in thermogenesis gene expression [195]. Quercetin and analogues treatment for 8 weeks induced browning of retroperitoneal and subcutaneous human WAT [42,196]. Rutin and luteolin support the results confirming the activation of browning and thermogenesis by AMPK/PGC1α pathway-mediated mechanism. In that way, they prevent high-fat diet (HFD)-induced overweight and insulin resistance [195]. Likewise, resveratrol and ECGC also have demonstrated their beneficial effect in the expenditure of energy [197]. One of the new approaches for obesity treatment could be dissipating energy by enhancement of BAT thermogenesis.

Reducing food intake is one of the advantageous effects that have been studied with flavonoids. The feeding behavior is controlled by the complex system that includes the activation of the central and peripheral nervous system, gastrointestinal tract, and other metabolic tissues, associated with the release of neurotransmitters, neuropeptides, and hormones [198]. The association of flavonoid and procyanidin has shown significant effect because these compounds induce satiety, satisfy hunger, or reduce craving urges to eat and, so, are useful in the prevention and treatment of overweight. Another study realized in treated rats with flavonoid-rich extract has shown that the food intake and weight gain were significantly less than the normal control because of generating satiety ability [199]. It is possible that flavonoids could modulate the signals of the agouti-related peptide (AgRP) given that agRP is co-expressed with neuropeptide Y and can stimulate appetite, hence causing weight gain [200].

On the other hand, rats fed anthocyanins have low plasma levels of cholesterol, TG, leptin, and resistin, even though in rat adipocytes anthocyanins increased the secretion of adiponectin and leptin, and the expression of PPARɣ, lipoprotein lipase (LPL), aP2, and UCP2 [201]. In addition, glucose also attenuated the FoxO1 factor, which produces a decrease in the expression of ATGL lipases, thus inhibiting TG lipolysis and plasma FFA levels. In white adipose tissue and skeletal muscle, it is speculated that the activation of AMPK (a protein also involved in autophagy) is responsible for these beneficial effects [202].

Recent investigations carried out on obese rats and 3T3-L1 preadipocytes have shown a decrease in lipid accumulation in adipocytes and liver, proposing that quercetin and one of its derivatives could modify unfavorable epigenomic profiles in obese rats [168]. Alocatechin or epigallocatechin have shown actions on nutritional status, with a decrease in BMI, fat mass, and inflammatory mediators. Quercetin in animal models shows clear evidence of a weak improvement in nutritional status and reduction of inflammation [203].

In a recent review, the authors further analyzed the role of quercetin and EGCG in obesity [109]. It was reported that both polyphenols showed anti-obesity effects in in vitro and animal models, but EGCG was more investigated than quercetin addressing this metabolic pathology. They have demonstrated to decrease adipogenesis and lipid accumulation by interfering with several enzymes and markers involved in lipid formation and breakdown in adipocytes. They have also shown to reduce adipocyte mass by inhibiting the proliferation and/or by promoting the apoptosis of adipocytes. They have consistently shown to decrease the inflammatory state of obese animals and, ultimately, contribute to improving glucose homeostasis and lipid profile. However, overall the anti-obesity effects in humans produced by quercetin are still unclear.

## 6. New Perspectives in the Flavonoids Study

Nowadays, the treatment of obesity has some gaps, and suitable management of the resources available is needed to obtain the best outcomes. The possibility of using natural products such as flavonoids in the treatment of obesity and, concurrently, improving the associated comorbidities is a resource that has not been widely examined yet. The development of effective and safe drugs against obesity is a therapeutic strategy where flavonoids should be included. The promising role of flavonoids in the treatment of obesity is described in preliminary reports, and subsequently, several in vitro and in vivo studies have documented their beneficial functions. However, clinical trials developed with these molecules are still scarce.

As we described above, flavonoid derivatives have activity on autophagy and inflammation, as well as anti-obesity actions demonstrated in a variety of animal models and in human studies.

The reduction of the inflammatory state and the changes in the autophagic flux in obesity are very important and, ultimately, contribute to improving other comorbidities related to glucose homeostasis and lipid profile. Certainly, flavonoids do not show potent effects after oral administration, generally because of the low bioavailability they present administered through this route. In addition, as described in Section 3, they suffer a fast metabolism in the body. Quercetin is absorbed ten times more efficiently than EGCG in humans, and both polyphenols have a half-life of 3.5 h [25], significantly longer than the general average for flavonoids. Although, it is unlikely that the differences in anti-obesity effects of both compounds, quercetin and EGCG, can be explained only by their pharmacokinetic profiles.

The flavonoids in blood maximal concentrations are generally very low. Administrated orally, except in special case, present a real plasma concentration less than 1 μM [204]. Flavonoids affect all stages of inflammatory processes even it is possible that the use of flavonoids in chronic and low inflammation was a good way of studying metabolic diseases. However, it is necessary to look for other routes of administration that improve their bioavailability. There are data demonstrating that some flavonoid glucuronides and glycosylated metabolites may show significant activity in the body via conjugation and deconjugation [205].

In summary, there are experimental data that support the relationship of flavonoids with obesity. So the cellular mechanisms leading to this beneficial effect should be further studied. On the other hand, obesity is directly associated with alterations in glucose metabolism and increased cardiovascular risk. In these cases, it is important to investigate the role that flavonoids play in autophagy, not only to improve the treatment of obesity but also because of the benefit it would have for those associated comorbidities.

## Figures and Tables

**Table 1:** Effects on autophagy in adipose tissue of obese models (human and rodents) and gene-modified animal models.

Reference	Model of Obesity	Parameters Studied	Effect in Autophagy
Obese human model			
Soussi, H. et al. (2015) [58]	Obese human (subcutaneous, white adipose tissue (WAT)	Increased DAPK2 and p62 mRNA, decreased LC3II expression	Decreased
Kovsan, J. et al. (2011) [59]	Obese human (omental and subcutaneous WAT)	Increased *ATG5*, LC3A and LC3B mRNA expression	Enhanced
Jansen, H. J. et al. (2012) [60]	Obese human (visceral and subcutaneous WAT, culture fat explant)	Increased *ATG7,* LC3II mRNA expression; IL1β, IL6, IL8 mRNA expression	Enhanced
Haim, Y. et al. (2015) [61]	Obese human (omental fat, explant WAT)	Increased *ATG5*, LC3II and E2F1 protein expression, decreased adiponectin	Enhanced
Xu, Q. et al. (2018) [62]	Obese human (abdominal WAT)	Increased *ATG5, ATG7 ATG12* expression, decreased HSL lipase expression	Enhanced
Nuñez, C.E. et al. (2012) [63]	Obese human (subcutaneous WAT)	Increased TNFα, IL-6, IL-1β, phospho-PERK, spliced-XBP1 and GRP78	Enhanced
Kosacka, J. et al. (2015) [64]	Obese and T2D patients (visceral and subcutaneous WAT)	Increased LC3 and *ATG5* mRNA, decreased p62 and mTOR protein levels.	Enhanced
Ost, A., et al. (2010) [65]	Obese and T2D human (subcutaneous WAT)	Decreased mTOR; enhanced LC3A	Enhanced
Obese animal model			
Jansen, H. J. et al. (2012) [60]	Obese leptin deficient (Lep^ob^) mouse (epididymal WAT)	Increased *Atg7*, LC3II mRNA expression and IL1β, IL6, IL8 mRNA expression	Enhanced
Lopez- Vicario, C. et al. (2015) [66]	HFD mice (epididymal WAT)	Increased *Atg12–Agt5* and LC3II levels; no change p62	Enhanced
Aijala, M. et al. (2013) [67]	Long-term fructose diet (WAT rat)	Decreased Atg7, LAMP2, MAP1, and LC3B	Decreased
Soussi, H., et al. (2015) [58]	HFD mice (isolated adipocytes and 3T3-L1 cells)	Increased DAPK2 and p62 mRNA, decreased LC3II expression	Decreased
Nuñez, C.E. et al. 2013 [63]	HFD mice (visceral adipose tissue)	Increased p62, Beclin and p62, decreased phospho-mTOR	Enhanced
Gene-modified animal models			
He, C. et al. (2013) [68]	Whole body Regular diet or HFD mice (Beclin2 ^+/−^)	Increased levels of brain cannabinoid 1 receptor, elevated food intake, insulin resistance, obesity	Suppressed
Yasuda- Yamahara, M. et al. (2015) [69]	Whole body HFD mice (Lamp2^y/^^−^)	Increased thermogenesis and energy expenditure, improved high-fat diet-induced obese diabetes	Suppressed
Liu, Y. et al. (2016) [70]	Whole body HFD mice (Bif1^−^^/^^−^)	Adipocyte hypertrophy, weight gain, downregulation expression of proteins of autophagy-lysosomal pathway, obesity, and insulin resistance	Suppressed
Pyo, J. O. et al. (2013) [71]	Whole body Regular diet mice (Atg5 overexpression)	Improved metabolism, increased insulin sensitivity, reduced blood levels of glucose	Enhanced
Lim, Y. M. et al. (2014) [72]	Whole body Bred with ob/ob mice (Atg7^+/−^)	Increased inflammasome activation, intracellular lipid content and insulin resistance after lipid loading	Suppressed
Singh, R. et al. (2009) [73] Zhang, Y. et al. (2009) [74]	WAT and 3T3-L1 preadipocytes Regular diet or HFD mice (Atg7^−^^/^^−^)	Inhibited lipid accumulation, decreased WAT mass, enhanced insulin sensitivity, decreased plasma concentrations of leptin but not adiponectin.	Suppressed
Singh, R. et al. (2009) [75] Shibata, M. et al. (2009) [76]	Liver Regular diet mice (Atg7^−^^/^^−^)	Increased hepatic lipid content [68] Decreased hepatic lipid content [69]	Suppressed
Kim, K. H. et al. (2013) [77]	Skeletal muscle HFD mice (Atg7^−^^/^^−^)	Decreased fat mass Protection against obesity and insulin resistance	Suppressed
Ebato, C. et al. (2008) [78] Jung, H. S. et al. (2008) [79]	Pancreas diabetic *db/db,* HFD or regular diet mice (β cells Atg7^−^^/^^−^)	Impaired glucose tolerance and reduced insulin secretion	Suppressed
Quan, W. et al. (2012) [80]	Pancreas Bred with ob/ob mice (β cells Atg7^−^^/^^−^)	ER stress, increased in beta cell death and accumulation of ROS, hyperglycemia and diabetes mellitus	Suppressed
Shigihara, N. et al. (2014) [81]	Pancreas HFD mice (β cells Atg7^−^^/^^−^, INS-1 cells)	Enhanced β-cell apoptosis, lower increased in β-cell mass and degenerative changes in pancreatic islets, obesity, elevated blood levels of glucose, glucose intolerance	Suppressed

**Table 2:** Summary of flavonoids effects on adipocytes and adipose tissue related with obesity (↓ = decreased, ↑ = increased).

Reference	Name of Flavonoids	Model of Study	Effects in Obesity
[151,152,153,154,155,156,157,158,159,160,161,162,163,164,165,166,167,168,169,170,171,172]	Flavonols: Quercetin	3T3-L1 adipocytes, HFD-induced obese mice	↓ PPARγ, C/EBPα, C/EBPα FABP4, aP2 and LPL genes, ↑ apoptosis, ↓ ERK and JNK phosphorylation, ↑ AMPKα1/SIRT1, ↓ E2F2 (Nrf2), ↑ c/EBPα, PPARγ, Caspase 3, Bax and Bak gene expression, ↓ number of macrophages, ↓ leptin, TNFα, NF-κB, NADPH oxidases, and antioxidant enzymes, ↓cholesterol metabolism and immune and inflammatory genes. Altered lipid expression genes: Fnta, Pon1, Pparg,, Aldh1b1, APOA4, Abcg5, Gpam, Acaca, Cd36, Fdft1, and Fasn
[154,173,174,175,176,177,178,179,180,181,182]	Flavon-3-ol: Epigallocathechin EGCG	3T3-L1 preadipocytes, adipocytes, immortalized brown preadipocytes	↓ cell viability, ↑ apoptosis. No effect on viability, ↑ S phase during differentiation, ↑ G2/M phase, ↑ Phosphorylation of AMPK, ↓ Phosphorylation of FOXO1, ERK1/2, Akt ↓ ACC, FAS and FOXO1 mRNA levels, No effect FOXO1, FOXO3 and SREBP-1c mRNA, ↓ Glut4 protein level, ↓ ROS ↓ PPARγ, C/EBPα, LXRα and SREBP-1c, FABP4 and ↑ β-Catenin mRNA levels ↓ Lipid accumulation, GPDH activity, ↑ HSL mRNA levels, ↓ HSL and resistin mRNA levels, ↑ UCP1 and UCP2 mRNA levels
[183,184,185,186]	Isoflavones: Genistein	3T3-L1 preadipocytes, HFD-induced obese mouse	↑ ROS release activated AMPK ↑ pro-caspase 3, Bax, cytochrome C, and PARP ↓ lipid accumulation, ↓ adipogenesis, ↑ apoptosis, ↓ lipogenic genes, (PPARγ) (C/EBPα), leptin and adiponectin. agonist/antagonist activity PPARγ
[156,187]	Flavons: Apigenin	HFD-induced obese mouse	↑ fatty acid oxidation, TAC, oxidative phosphorylation, electron transport chain and cholesterol expression of genes, ↓ lipogenic and lipolytic genes expression, ↓ triglyceride and cholesterol enzymes, ↓ PPARγ, ↓ oxidative stress
[169,188]	Anthocianins: Cyanidin	3T3-L1 cell, HFD-induced obese mouse	↓ adipocyte life cycle, ↓ adipocyte proliferation, ↓ adipogenesis ↓ lipolysis and apoptosis induction. ↑ leptin, resistin. Not change cholesterol, triglycerides, (MCP-1)
